# Bioaccumulation dynamics of perfluorooctane sulfonic acid (PFOS) and a binary mixture of PFOS and perfluorohexane sulfonic acid (PFHxS) in northern bobwhite (*Colinus virginianus*): a mini review

**DOI:** 10.1007/s10646-026-03160-2

**Published:** 2026-09-30

**Authors:** Kenneth N. Kikanme, Adcharee Karnjanapiboonwong, Nicole M. Dennis, Rameshkumar Angappan, Efe Jeffery Isukuru, Christopher J. Salice, Todd A. Anderson

**Affiliations:** 1 Department of Environmental Toxicology, Texas Tech University, Lubbock, TX USA; 2 AXIS Environmental Intelligence Group, LLC, Phoenix, AZ USA; 3 Environmental Science and Studies, Towson University, Towson, MD USA

**Keywords:** Bird, PFAS, PFOS, Bioaccumulation

## Abstract

Per- and polyfluoroalkyl substances (PFAS) are persistent environmental contaminants with significant bioaccumulation potential and toxicological concerns. Specifically, perfluorooctane sulfonic acid (PFOS) has been shown to reduce growth, reduce hatching success and decrease hatchling survivability, and arrest developmental cycles in birds. While the transfer of PFAS from exposed adult birds to their offspring is recognized, the routes of maternal transfer are not yet fully elucidated. Therefore, this mini review synthesizes previously collected. PFAS tissue residue data from a series of chronic toxicity studies in Northern bobwhite (NOBO) conducted in our laboratory, integrating findings from adult tissues, whole eggs, the chorioallantoic membrane (CAM), and hatchling tissues. Our goal was to provide a better understanding of tissue-specific PFAS bioaccumulation in the adult, and maternal transfer to the developing chick. The available data revealed that the liver serves as the primary site for PFAS bioaccumulation in adult and hatchling NOBO, following the trend: adult liver > adult brain > post-hatch CAM > whole egg > hatchling liver > hatchling brain. Hatchling tissues exhibited lower PFAS concentrations in brain and in liver compared to those same tissues in adult females; hatchlings received PFAS exposure only through maternal transfer during embryonic development. The data from our studies further highlight that assessing the post-hatch CAM, rather than the whole egg, is important because it serves as a direct link between the embryo and the egg’s internal environment, thus reflecting a PFAS burden that is available to the developing chick; whole egg measurements capture total PFAS deposited by the female NOBO into the egg. Findings from controlled PFAS exposures to birds conducted in our laboratory indicate that maternal PFAS burdens influence hatchling exposure and contribute to PFAS bioaccumulation patterns.

## Introduction

Per- and polyfluoroalkyl substances (PFAS) are highly persistent chemicals with slow rates of thermal degradation and biotransformation. Due to their extensive applications across various industries including manufacturing, healthcare, construction, food packaging, and consumer goods, PFAS are widely detected in environmental media such as soil, air, and water (USEPA 2024a). Among these compounds, perfluorooctane sulfonic acid (PFOS) is one of the most studied and historically utilized (USEPA 2024b). Since 2009, PFOS has been listed under Annex B of the Stockholm Convention and restricted. More recently (2022), perfluorohexane sulfonic acid (PFHxS), along with its salts and related compounds, was added to Annex A of the Convention, signaling a shift toward its elimination (Stockholm Convention 2024).

In the United States, PFOS has been gradually replaced with alternative PFAS such as perfluorobutane sulfonic acid (PFBS), as well as fluorinated ethers like GenX (Li et al. 2020); these alternatives were selected primarily due to their shorter carbon chains. However, despite its phase-out, environmental concentrations of PFOS have shown declining trends in many biotic and abiotic compartments (Iyer et al. 2026; Sun et al. 2026), but remain high in specific regions and matrices. East et al. (2025) found that for soils, PFOS remains the most prevalent and highest-concentration PFAS in aqueous film forming foam (AFFF)-affected U.S. Air Force sites, accounting for approximately 82% of site-specific PFAS mixtures, followed by PFHxS, perfluorooctanoic acid (PFOA), perfluorooctane sulfonamide (PFOSA), and perfluorohexanoic acid (PFHxA). It is important to note that these findings are based on targeted measurements and may not account for unidentified or unquantified PFAS present at these sites. In a broader context, Moghadasi et al. (2023) observed that while PFAS concentrations in European soils generally decrease with distance from point sources, a significant portion exceeds 5000 ng/kg, posing a risk of water contamination above drinking water guidelines and suggesting a lag in the response to its removal (Brusseau et al. 2020; Buytaert et al. 2023). Consequently, PFOS readily enters organisms and interacts with blood and tissue proteins, contributing to its widespread detection in wildlife (Cao and Ng 2021).

Growing evidence highlights the association between specific long chain PFAS exposures and their binding affinity to tissue-specific proteins, resulting in bioaccumulation across diverse adult avian species and tissues. Nordén et al. (2013) quantified PFOS concentrations in the yolk of cormorants (*Phalacrocorax* sp.) and herring gulls (*Larus argentatus*) at 1,506 ng/g wet weight (ww) and 589 ng/g ww, respectively, but slightly lower concentrations in liver tissues (583 ng/g ww in cormorants and 508 ng/g ww in herring gulls). Similarly, Sinclair et al. (2006) documented PFOS accumulation in the livers of 10 waterfowl species, with concentrations ranging from 11 to 882 ng/g ww; PFHxS remained undetectable in all liver samples analyzed. Holmström and Berger (2008) observed tissue-specific variations in PFOS levels with concentrations of 121 ng/g ww in the liver and 325 ng/g ww in the eggs of adult common guillemots (*Uria aalge*). Newsted et al. (2007) reported significant PFOS bioaccumulation in the liver and serum of mallard ducks (*Anas platyrhynchos*) and Northern bobwhite quail (*Colinus virginianu*s) following dietary exposure to 10 mg PFOS/kg feed in laboratory studies. Gebbink and Letcher (2012) highlighted PFOS as the dominant contributor to the total concentration of various perfluoroalkyl sulfonic acids (PFSA), accounting for over 88% of the total in Great Lakes herring gulls.

In birds, the liver is essential for digestion and metabolic processes, playing a key role in the synthesis, storage, and regulation of lipids, carbohydrates, and proteins (Denbow and Whittow 2015; Zaefarian et al. 2019). Robuck et al. (2020) indicated that PFOS may interfere with lipid metabolism by suppressing gene expression, thereby potentially influencing lipid levels indirectly. Given the propensity of PFAS to accumulate in liver, particularly PFOS, raises concerns about the potential for chemical-induced metabolic disruption in avian species.

Some studies have evaluated the relationship between PFOS and PFHxS concentrations in avian reproductive and internal tissues. Holmström and Berger (2008) reported elevated PFOS levels (309 ng/g ww) in the livers of common guillemot (*Uria aalge*) chicks, while PFHxS was below detection in both chick and adult livers, and only marginally present in eggs (1.5 ng/g ww). Similarly, Yoo et al. (2008) confirmed higher PFOS levels in the egg yolks of little egrets (*Egretta garzetta*), parrotbills (*Paradoxornis webbiana*), and little ringed plovers (*Charadrius dubius*) compared to PFHxS. Holmström and Berger also identified PFOS and perfluorocarboxylic acids (PFCAs) as primary markers of PFAS body burden in the eggs and organs of common guillemots and their chicks. Likewise, Robuck et al. (2021) documented significant PFAS accumulation across multiple organs in young seabirds. More recently, Gao et al. (2023) demonstrated that in chickens, PFAS exposure resulted in greater concentrations of total PFAS in yolk tissues compared to other organs, with PFOS identified as the dominant compound.

Age-related differences in PFOS bioaccumulation have also been documented. For instance, Dauwe et al. (2007) reported that young great tits (*Parus major*) under one year of age exhibited significantly higher liver PFOS concentrations compared to older birds, although no sex-based differences in liver PFOS accumulation were noted. This age-related pattern supports the notion of maternal transfer, suggesting that chicks may accumulate PFOS *in ovo* prior to hatching.

Evidence of PFOS bioaccumulation extends beyond liver and reproductive tissues, with growing reports of its presence in avian brain tissues an emerging area of concern given the neurobehavioral roles of brain regions such as the dorsal ventricular ridge (DVR), which lies outside the blood-brain barrier (BBB) (Olivero-Verbel et al. 2006; Nomura and Izawa 2017; Kikanme et al. 2025a). Birds rely heavily on foraging and communication behaviors regulated by these regions, making birds potentially more vulnerable to PFAS-induced neurotoxicity. Verreault et al. (2005) measured elevated PFOS concentrations in the brain, liver, and eggs of glaucous gulls (*Larus hyperboreus*), however, PFHxS concentrations were consistently below detection limits. Similarly, Robuck et al. (2021) observed a higher frequency of PFOS detection compared to PFHxS in the brain and liver tissues of juvenile seabirds.

These results reaffirm the pervasive nature of PFOS and its ability to bioaccumulate in birds across a broad range of species, tissues, and ages (adult and chicks). However, they also highlight a potential knowledge gap in our understanding of PFAS bioaccumulation pathways during avian embryonic development. While previous studies have primarily focused on the yolk and albumin as the main reservoirs for maternal transfer, the chorioallantoic membrane (CAM) may also serve as an additional route for PFAS transfer to embryonic tissues (Kikanme et al. 2025b). Eggshell samples contain remnants of the CAM, which typically remains attached to the shell after hatching. The CAM forms during embryonic development through the fusion of the chorion and allantois. It is a highly vascularized structure that develops early, prior to organogenesis, and continues to mature throughout incubation. Functionally, the CAM plays a critical role in gas exchange, facilitates nutrient transfer from the yolk, and serves as a site for waste sequestration (Cobb et al. 2003). Despite its biological relevance, the CAM has been overlooked in PFAS exposure studies, with most prior work focusing instead on egg contents and hatching tissues. Because the CAM receives a direct blood supply from the developing embryo, it may reflect contaminant exposure during critical periods of development (Bargar et al. 2003; Pepper et al. 2004).

The present manuscript is a mini review that synthesizes and integrates previously published PFAS tissue-residue data generated from a series of controlled PFAS chronic exposure studies in NOBO by our research group. Building on the results of our progressive research, this review collates results from multiple investigations into a single framework to evaluate patterns of PFAS distribution among adult male and female liver and brain tissues, whole eggs, the CAM, and hatchling liver and brain tissues. By bringing these datasets together and comparing our results to other studies on PFAS in birds (when available), this mini review provides an assessment of maternal PFAS transfer and tissue-specific bioaccumulation dynamics in NOBO and highlights the potential role of the CAM as an informative matrix for evaluating embryonic PFAS exposure. This synthesis contributes to a broader understanding of the environmental and toxicological implications of PFAS exposure in avian wildlife.

## Materials and methods

### Data

This manuscript presents a synthesis of empirical data from multiple trials, conducted in our laboratory, investigating the chronic toxicity of PFOS and PFOS: PFHxS in exposed NOBO. The studies span a range of experimental conditions, including investigations of tissue-specific avian chronic toxicity values, potential sex-specific differences in bioaccumulation, and maternal transfer. Despite differences in study objectives and biological matrices, the datasets are analytically comparable because they employed similar extraction and quantification approaches, including QuEChERS-based extraction methods, the use of isotopically labeled surrogates and internal standards, and LC-MS/MS quantification under comparable conditions. However, some variability in extraction volumes, sample masses, and tissue-specific processing protocols exist, which may influence absolute concentration comparisons across studies. As such, the integrated dataset is interpreted with an emphasis on relative distribution patterns and tissue-specific trends rather than quantitative equivalence across all matrices. Details of the study designs, experimental setups, data collection protocols, and statistical comparisons were presented in the original publications (Dennis et al. 2020; 2021; Kikanme et al. 2025a; 2025b).

There were three main groups of experimental birds: control (unexposed), PFOS-exposed (single compound), and PFOS: PFHxS-exposed (binary mixture). In the PFAS-exposed groups, data for our bioaccumulation dynamics analyses were from adult NOBO exposed orally via drinking water to (analytically verified) 20 ng/mL of PFOS or PFOS: PFHxS, with an exposure duration of 60 days.

NOBO began laying eggs approximately 15 days after being exposed to increased light conditions. We collected the eggs as they were laid; every 10 days, a set of eggs was either incubated or archived for residue analysis. Chicks that hatched from incubated eggs were raised for 21 days. We recognize that exposure duration and laying sequence may have some influence on PFAS deposition into eggs and subsequent residue analyses.

**Fig. 1:**
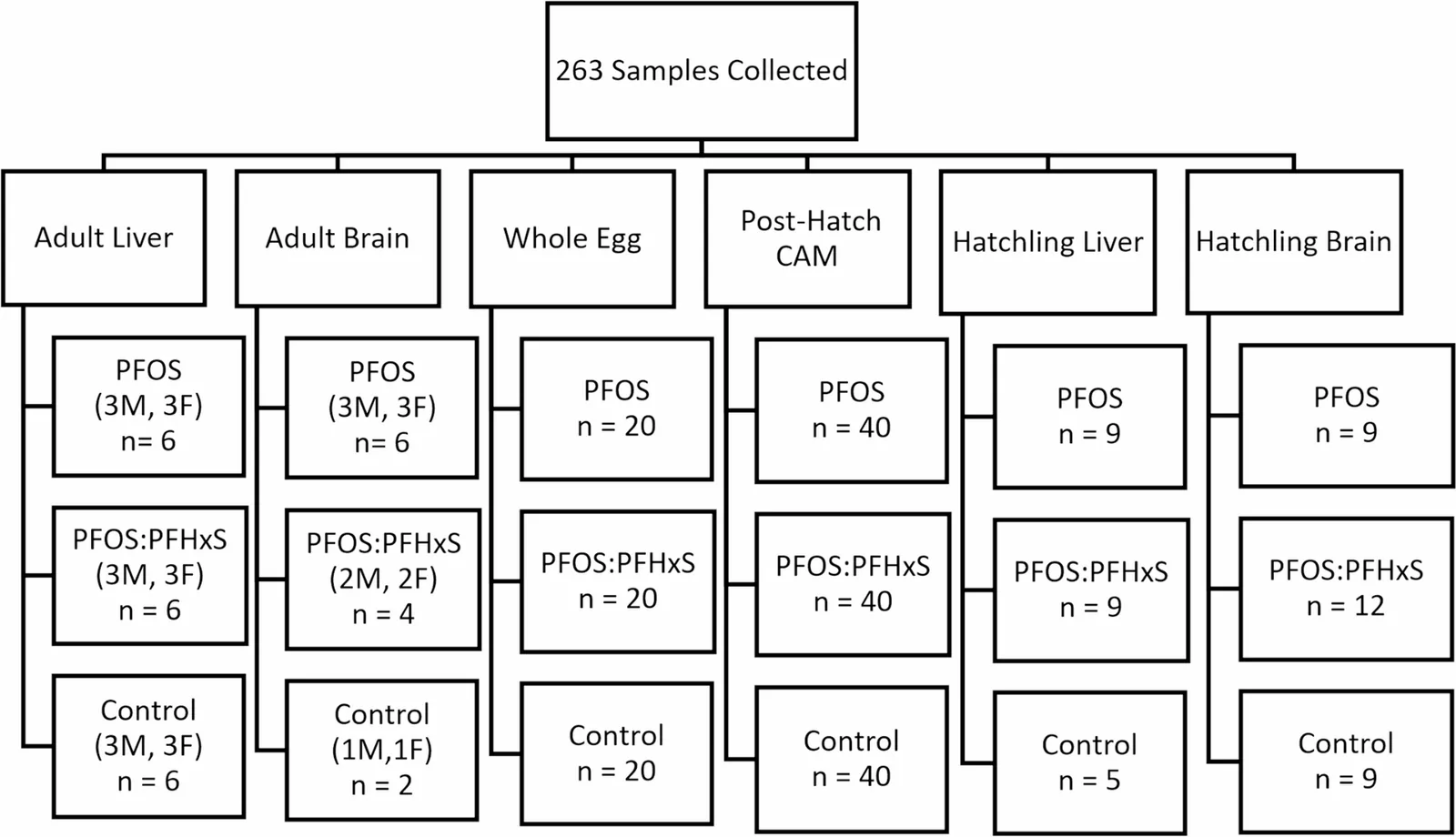
Experimental outline for samples from controlled laboratory exposure studies with PFAS in NOBO. M = adult male. F = adult female

A total of 263 samples were collected from PFOS and PFOS: PFHxS mixture laboratory NOBO exposure studies to evaluate the distribution of PFOS and PFHxS across various NOBO tissues (Fig. 1). All animal experiments conducted in the original studies adhered to the ethical guidelines established by the Institutional Animal Care and Use Committee (IACUC) of Texas Tech University, Protocol 16108-12. This study was conducted and reported in accordance with the Animal Research: Reporting of In Vivo Experiments (ARRIVE) guidelines.

### PFAS determinations

Sample preparation across studies was based on modified QuEChERS extraction approaches tailored to each tissue type. Specifically, liver and egg homogenates were extracted using QuEChERS methods with salt partitioning and isotopically labeled surrogate standards, while brain and CAM samples were processed using similar solvent extraction procedures with minor modifications in sample mass, extraction volumes, and cleanup steps to accommodate tissue-specific properties.

To ensure analytical reliability across matrices, all methods incorporated isotopically labeled surrogate and internal standards, procedural blanks, and recovery assessments. The use of isotope dilution and matrix-matched quality control approaches minimized potential matrix-induced bias and supports comparability of PFAS measurements across tissues. Accordingly, results are interpreted with consideration of potential matrix-specific variability, with emphasis on relative distribution patterns rather than cross-matrix quantitative equivalence. We did have the advantage of knowing exactly what the birds were exposed to, which removes some of the uncertainty in PFAS identification that can be problematic in forensic PFAS exposure investigations on wild-caught birds.

## Results and discussion

Previous studies in our lab (Dennis et al. 2020; 2021; Kikanme et al. 2025a; 2025b) evaluated the presence of statistical differences in the bioaccumulation, toxicological effects, and maternal transfer of PFAS in adult NOBO and their offspring following chronic exposure. Control birds (unexposed) showed no detectable levels of PFOS or PFHxS across all NOBO tissues, except for trace amounts in CAM and brain tissue (Table 1). This is likely the result of NOBO being sourced from pen-raised environments where exposure to various PFAS through dietary items is likely.

**Table 1:** Mean concentrations of PFOS and PFHxS across adult and hatchling tissues (liver, brain whole egg, and CAM) in Northern Bobwhite following 90-day trial (60-day oral exposure)

Exposure Group	ADI^1^	Analyte	Liver (ng/g ± SE)		Brain (ng/g ± SE)		Whole Egg (ng/g ± SE)	CAM (ng/g ± SE)	Hatchling (ng/g ± SE)	
			Male	Female	Male	Female			Brain	Liver
Control	-	PFOS	ND	ND	ND	ND	ND	3.5 ± 1.7	0.8 ± 0.5	ND
	-	PFHxS	ND	ND	ND	ND	ND	-	0.1 ± 0.02	ND
PFOS	2.45	PFOS	565 ± 24	226 ± 8	102 ± 15	15 ± 5.1	92 ± 14	142 ± 16	4.0 ± 0.5	50 ± 5.1
PFOS: PFHxS^2^	3.1	PFOS	463 ± 43	325 ± 111	108 ± 16	11 ± 5.9	50 ± 5.1	93 ± 9	1.9 ± 0.5	46 ± 3.5
		PFHxS	251 ± 44	138 ± 40	29 ± 8.3	15 ± 10	36 ± 4.1	51 ± 19	0.9 ± 0.3	9.5 ± 5.7

^1^Average Daly Intake (ng/g body weight/day) is based on either PFOS, or both PFOS and PFHxS

^2^Analytically measured ratio was 1.2:1

SE = Standard Error

Statistically significant sex differences within the Exposure Group

Statistically significant difference (whole egg vs. CAM) within the Exposure Group

### PFAS concentrations: adult NOBO, eggs, and CAMs

**Fig. 2:**
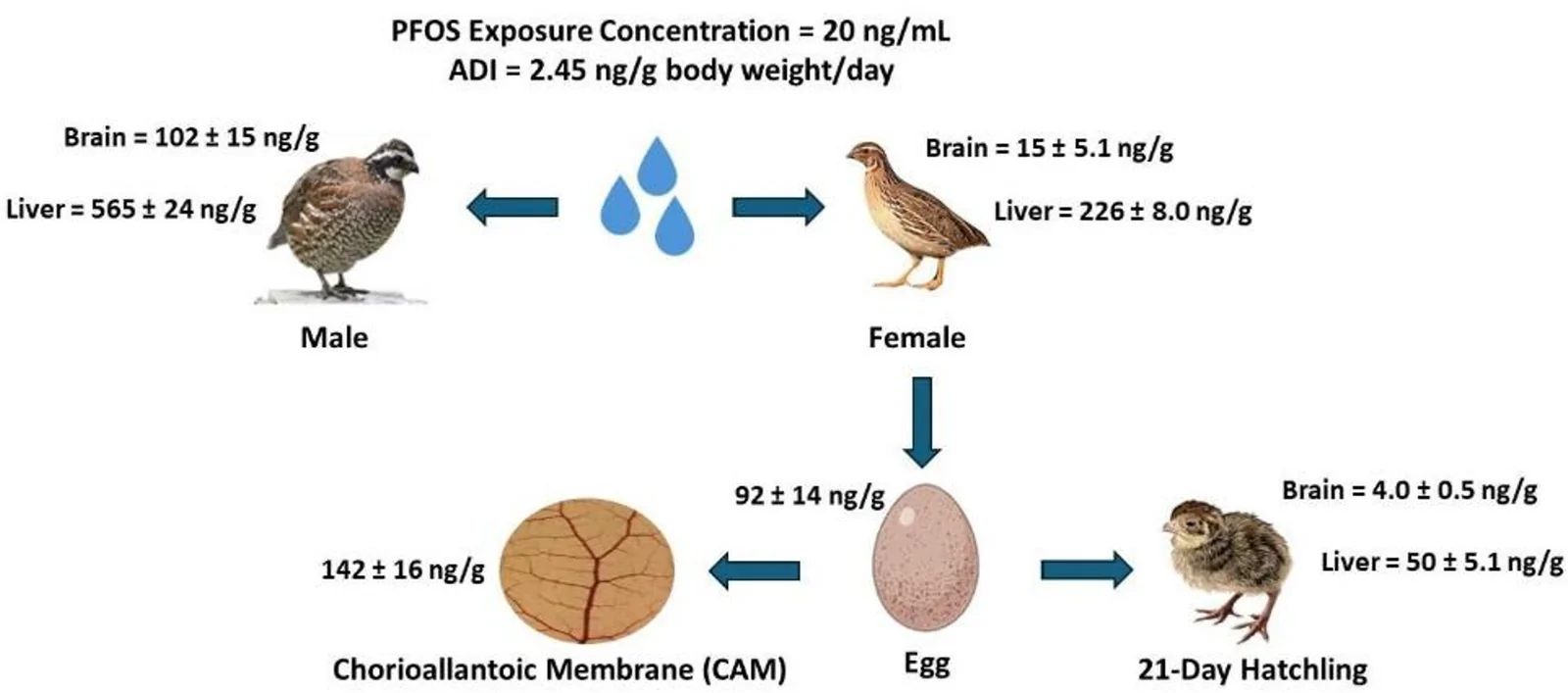
PFOS concentrations in various tissues following adult exposure to PFOS via drinking water for 90 days

**Fig. 3:**
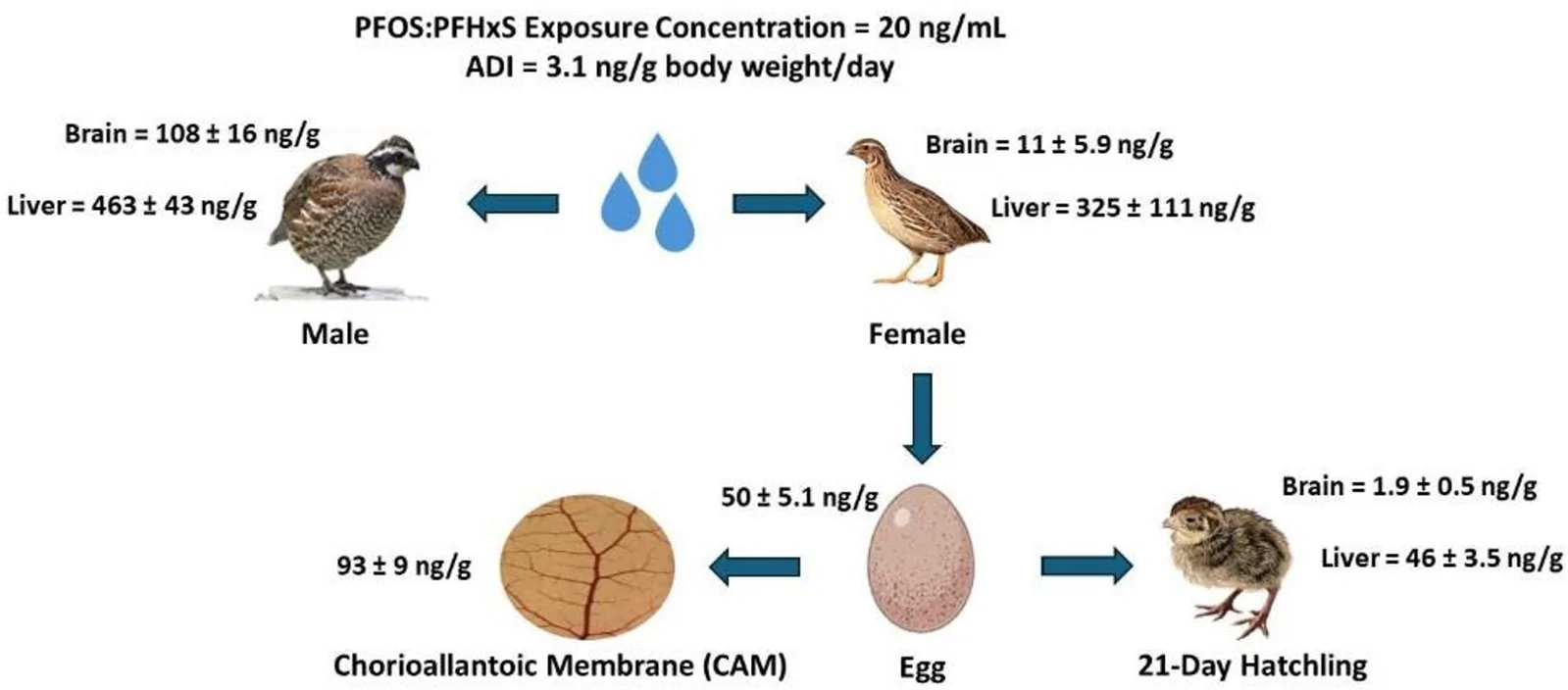
PFOS concentrations in various tissues following adult exposure to a binary mixture of PFOS: PFHxS via drinking water for 90 days

**Fig. 4:**
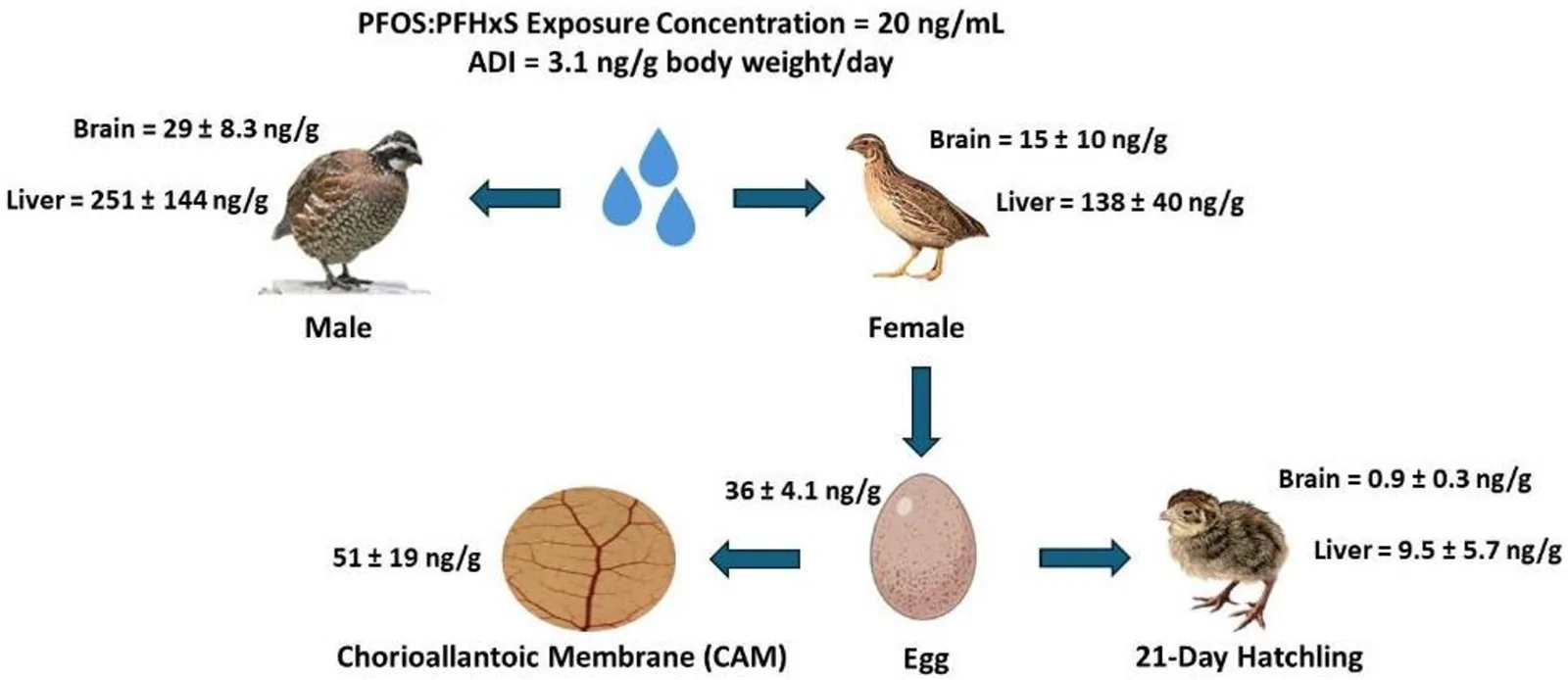
PFHxS concentrations in various tissues following adult exposure to a binary mixture of PFOS: PFHxS via drinking water for 90 days

PFOS was determined in all experimental groups, whereas PFHxS was determined only in the binary mixture group, as shown in Table 1. PFOS had the highest concentration in NOBO tissues (Fig. 2) compared to the control group, particularly in the liver (565 ng/g ww in males). PFOS concentrations from the PFOS: PFHxS binary mixture exposure (Fig. 3) were 463 ng/g in livers of male NOBO and 325 ng/g ww in the livers of female NOBO. PFHxS concentrations in livers were statistically lower in males (*p* = 0.029) but not females (*p* = 0.21) in the PFOS: PFHxS binary mixture exposure (Fig. 4) compared to PFOS concentrations. These results suggest preferential accumulation of PFOS in the liver, possibly due to its affinity for liver-binding fatty acid proteins (LBFAP) and the absence of oviposition in male NOBO. Similar concentrations of PFOS have also been reported in males (390 ng/g ww) and females (385 ng/g ww) of common cormorants (*Phalacrocorax carbo*), but with no significant differences observed between sexes (Kannan et al. 2002). PFOS concentrations in the brain (from PFOS-only exposure and from the PFOS: PFHxS binary mixture exposure) were statistically higher in male NOBO compared to female NOBO (Kikanme et al. 2025a). Collectively, these findings align with Yoo et al. (2009), which reported higher PFOS accumulation in the liver than in the brain of white leghorn chickens (*Gallus gallus*) exposed to PFOS.

PFOS concentrations in whole eggs laid by female NOBO and post-hatch CAMs (from PFOS-only exposure, Table 1and Fig. 2) were slightly higher (*p* = 0.024) in post-hatch CAMs (142 ng/g ww) than whole eggs (92 ng/g ww), suggesting that PFOS was adsorbed or more retained in the post-hatch CAM compared to the whole egg. However, previous studies by Newsted et al. (2007) and Holmstrom and Berger (2008) reported that PFOS predominantly accumulated in the egg yolk, attributed to its association with very low-density lipoproteins (VLDLP). Similarly, Vicente et al. (2015) demonstrated that PFAS exhibited a stronger binding affinity for egg-yolk proteins compared to albumen proteins. These findings highlight the need for further investigations into the roles of albumen, egg yolk, and CAMs in PFAS accumulation and maternal transfer to hatchlings.

PFOS concentration patterns (PFOS: PFHxS binary mixture exposure, Fig. 3) were statistically similar to PFOS-only exposure, but with a trend towards relatively lower concentrations in NOBO tissues. Liver concentrations of PFOS trended lower for male NOBO (463 ng/g ww vs. 565 ng/g ww) but higher in female NOBO (325 ng/g ww vs. 226 ng/g ww) exposed to the binary mixture compared to PFOS-only exposure; however, neither pattern was statistically significant. PFOS brain concentrations for the binary mixture exposure were similar for male NOBO (108 ng/g ww) and female NOBO (11 ng/g ww) when compared by sex to the PFOS-only exposure (Table 1). In whole eggs and post-hatch CAMs, PFOS concentrations for the binary mixture exposure (50 ng/g ww and 93 ng/g ww, respectively) were statistically lower than PFOS concentrations in whole eggs laid by female NOBO (92 ng/g ww; *p* = 0.0098) and post-hatch CAMs (142 ng/g ww; *p* = 0.013) from the PFOS-only exposure.

PFHxS liver concentrations for the binary mixture exposure (Table 1and Fig. 4) were 251 ng/g ww (male NOBO) and 138 ng/g ww (female NOBO); these concentrations in livers of male and female NOBO trended lower than PFOS concentrations in their respective counterparts (sex) from the PFOS-only and the binary mixture exposures (statistically so for male NOBO, *p* = 0.029). PFHxS brain concentrations for the binary mixture exposure were 29 ng/g ww (male NOBO) and 15 ng/g ww (female NOBO). PFHxS concentrations in whole eggs and CAMs for the binary mixture exposure trended lower than PFOS concentrations in those same tissues regardless of exposure (PFOS-only or PFOS: PFHxS binary mixture; *p* = 0.04 for whole eggs and *p* = 0.049 for CAMs).

### PFAS concentrations in 21-d hatchlings

The livers of NOBO hatchlings had similar PFOS concentrations whether the exposure to their parents occurred as PFOS-only (Table 1; Fig. 2) or as a binary mixture (PFOS: PFHxS, Table 1; Fig. 3); a similar pattern was observed for brain concentrations of PFOS in hatchlings. PFHxS concentrations (parental binary mixture exposure, Fig. 4) in hatchling livers (9.5 ng/g ww) and brains (0.9 ng/g ww) trended lower than PFOS concentrations in those same tissues regardless of parental exposure (PFOS-only or PFOS: PFHxS binary mixture); statistically lower (*p* = 0.0001) in liver.

### PFAS tissue distribution trends

Of the tissues that were examined, the liver exhibited the highest PFAS accumulation across all exposure groups (Figs. 2 and 3, and 4). In wildlife tissues, PFAS accumulation and elimination are influenced by various proteins, including fatty acid binding proteins (FABPs), organic anion transporters (OATs), albumin, vitellin, and high-density lipoproteins (HDL) (Bangma et al. 2022; Jouanneau et al. 2021; Wang et al. 2019). Across all exposure groups, livers and brains of male NOBO trended higher for PFAS accumulation than those same tissues in female NOBO, suggesting the potential for sex differences in PFAS bioaccumulation. Furthermore, in both males and females, brain tissue trended lower for PFAS accumulation than liver tissue in PFOS-only and PFOS: PFHxS-exposed groups, possibly due to the protective effect of the blood-brain barrier (Cao and Ng 2021; Kikanme et al. 2025a).

Our results suggest that mixture exposure may produce interactive effects, altering tissue concentrations compared to single PFAS exposures and highlighting the complex interactions between these chemicals in biological systems. In adult liver and brain tissues from the PFOS: PFHxS binary mixture exposure, where PFOS accumulation trended higher in males and females compared to PFHxS, the presence of PFHxS in the binary mixture appears to influence PFOS bioaccumulation in both sexes. A mechanism potentially responsible for this pattern is competitive binding to tissue proteins, such as albumin, liver fatty acid–binding proteins, and protein transporters, which have high affinity for PFAS (Zhao et al. 2023; Wen et al. 2019). Previous studies have shown that the accumulation of perfluoroalkyl acids (PFAAs) in tissues, including the brain, increases with perfluorinated carbon chain length, likely due to stronger hydrophobic interactions that enhance binding of longer-chain compounds (Wen et al. 2017). In this context, longer-chain PFAS, such as PFOS, may preferentially occupy high-affinity binding sites; however, longer-chain PFAS, such as PFHxS, may still compete for these sites, thereby altering PFOS distribution and tissue partitioning.

The accumulation of PFOS in whole eggs is influenced by PFAS binding to phospho- and lipoproteins synthesized in female bird livers, which are then transported via the bloodstream to the ovary and deposited in eggs (Ricolfi et al. 2024). Post-hatch CAMs trended towards higher accumulation of PFOS alone and PFOS in the PFOS: PFHxS mixture compared to whole eggs. This suggests that PFAS distribution in developing embryos is influenced by the blood supply, the affinity of different tissues for these compounds, and the rate of depuration. Also, PFOS alone and PFOS in the binary mixture trended towards higher accumulation in the CAM and whole egg compared to PFHxS, which may indicate differences in protein binding.

Avian CAMs have a diverse array of proteins, including structural and binding-associated proteins such as integrins and histones (Ahmed et al. 2022), some of which have been shown to interact with PFAS compounds (Tsai et al. 2024; Mei et al. 2024). Although protein composition may vary across species, the abundance of proteinaceous binding sites in the CAM, coupled with its high vascularization, may enhance PFAS partitioning into this membrane relative to the whole egg. This combination of protein-binding potential and continuous exposure via embryonic circulation likely contributes to the CAM acting as a reservoir for PFAS, distinguishing it from whole egg and highlighting its possible relevance as a sensitive matrix for assessing PFAS bioaccumulation during development.

### Comparison of PFAS concentrations: adult females vs. hatchlings

Not surprisingly, PFAS concentrations were notably higher in livers of female NOBO compared to livers of 21-d hatchlings, likely the result of direct oral exposure of adults over 60 days in contrast to the lack of post-natal exposure for hatchlings; in our trials, PFAS concentrations in hatchling livers are the result of maternal transfer during embryonic development. Specifically, PFOS concentrations in hatchling livers were approximately 4.5 times lower than in the liver of adult female birds from the PFOS-only exposure group. In the binary mixture exposure group, PFOS and PFHxS were lower in hatchling livers (7X and 14X lower, respectively), compared to the liver of female NOBO.

In the hatchling brain, PFOS concentrations were approximately 3.8X lower than PFOS in the adult female brain (PFOS-only exposure group). In the binary mixture exposure group, PFOS and PFHxS were lower in hatchling brains (5X and 16X lower, respectively), compared to brains of female NOBO.

### Maternal transfer dynamics

Residue data indicated that fate and transport of PFOS and PFHxS in biological tissues may be facilitated by protein-rich sites in the egg, CAM, and tissues. Specifically, PFOS may exhibit high transfer from maternal tissues to whole egg and high accumulation in the post-hatch CAM, because of its strong affinity for proteins in biological tissues and egg compartments. In the binary mixture exposure, PFOS concentrations in maternal tissue, whole egg, post-hatch CAM, and hatchling tissues trended consistently higher than PFHxS in those same tissues. The presence of PFHxS in the binary mixture appeared to reduce PFOS accumulation in maternal tissues, whole eggs, post-hatch CAMs, and hatchling tissues.

Bioaccumulation factors (BAFs; PFAS tissue concentration/PFAS intake concentration) were interpreted as follows: BAF > 1 indicates bioaccumulation, BAF = 1 indicates bioindication, and BAF < 1 indicates no bioaccumulation (Proc et al. 2021). In this study, we observed the highest BAFs for PFOS in liver (Table 2). Further, BAFs tended consistently higher for male birds vs. female birds (livers). The quantity of water consumed by breeding pairs had been monitored to determine PFAS intake. While the measured ADI during the exposure was presented as the same for the breeding pairs, it is likely that females consume more water during egg production and thus have a higher ADI than males, making the apparent higher BAFs in males even more striking.

**Table 2:** Bioaccumulation factors (BAF) for PFOS and PFHxS in adult and hatchling tissues (brain, liver, whole egg, and CAM) of Northern Bobwhite following 90-day trial (60-day oral exposure)

Exposure Group	Analyte	Liver		Brain		Whole Egg	CAM	Hatchling	
		Male	Female	Male	Female			Brain	Liver
PFOS	PFOS	2.6	1.0	0.5	0.1	0.4	0.6	0.02	0.2
PFOS: PFHxS	PFOS	1.7	1.2	0.4	0.04	0.2	0.3	0.01	0.2
	PFHxS	0.9	0.5	0.1	0.05	0.1	0.2	0.003	0.03

BAF = PFAS tissue concentration (ng/g) / PFAS adult intake concentration (ng/g)

In contrast, BAFs for PFOS alone and PFOS as part of a binary mixture in other NOBO tissues, such as brain, post-hatch CAM, whole egg, and hatchlings (brain and liver), were < 1, indicating no bioaccumulation. Notably, a BAF = 1.0 in the liver of female NOBO under PFOS-only exposure strongly suggests a state of equilibrium between PFOS intake and clearance. This equilibrium is characteristic of bioindicator behavior, where the tissue concentration reflects exposure without significant buildup (Proc et al. 2021). In contrast, the liver BAF = 2.6 for male NOBO under the same exposure condition further supports the presence of sex-specific toxicokinetics.

## Conclusions

PFOS concentrations in tissues (binary mixture exposure) exhibited a distribution pattern similar to PFOS-only exposure, whereas PFHxS in the binary mixture exposure showed the lowest accumulation in most tissues, particularly in the liver and brain of hatchlings. The liver appeared to be a major site of PFAS accumulation since high concentrations of PFOS and PFHxS were observed. Bioaccumulation of PFOS and PFHxS in whole egg and post-hatch CAM highlighted the maternal transfer routes where PFAS stored in female NOBO liver and other tissues were mobilized into the whole egg and CAM during the breeding cycle. Notably, the post-hatch CAM showed higher PFOS accumulation than the whole egg, suggesting its potential use as a biomarker for embryonic PFAS exposure. Male NOBO appeared to accumulate more PFOS in the liver and brain compared to female NOBO, underlining the need to consider sex-specific patterns in PFAS toxicity and distribution. Additionally, tissue data reveal some crude relationships in PFAS concentrations between female NOBO and hatchling tissues. Finally, the elevated concentrations of PFOS and PFHxS in the liver and brain of male NOBO, likely due to fewer effective PFAS elimination routes, may pose a higher risk of organ injury compared to female NOBO. These findings highlight the importance of incorporating sex-specific factors into PFAS risk assessments.

Future work should focus on elucidating the mechanistic drivers of PFAS distribution across tissues, particularly under binary-mixture exposure scenarios and in the presence of competitive interactions among PFAS. Additionally, further evaluation of the CAM biochemical composition and binding capacity could help strengthen our understanding of its utility as a sensitive indicator of embryonic PFAS exposure. 

## Data Availability

No datasets were generated or analysed during the current study.
